# Role of oxide support in Ni based catalysts for CO_2_ methanation

**DOI:** 10.1039/d1ra02327f

**Published:** 2021-05-14

**Authors:** Ye Hwan Lee, Jeong Yoon Ahn, Dinh Duc Nguyen, Soon Woong Chang, Sung Su Kim, Sang Moon Lee

**Affiliations:** Department of Environmental Energy Engineering, Graduate School of Kyonggi University 94-6 San, Iui-dong, Youngtong-ku Suwon-si Gyeonggi-do 442-760 Korea; Department of Environmental Energy Engineering, Kyonggi University 94-6 San, Iui-dong, Youngtong-ku Suwon-si Gyeonggi-do 442-760 Korea sskim@kyonggi.ac.kr leesangm@kyonggi.ac.kr

## Abstract

The CO_2_ methanation reaction of reduced and unreduced Ni based CeO_2_, Al_2_O_3_, TiO_2_ and Y_2_O_3_ supported catalysts was investigated. The Ni/CeO_2_ and Ni/Y_2_O_3_ catalysts exhibited similar CO_2_ conversions at all reaction temperatures. The catalysts were studied by X-ray diffraction (XRD), H_2_ chemisorption, H_2_ temperature-programmed reduction (TPR), and *in situ* diffuse reflection infrared Fourier transform spectroscopy (DRIFTS); the results suggested that the reducibility of both metal and support at low temperature, strong metal support interaction and small Ni particle size are important factors for low-temperature CO_2_ methanation. Based on the DRIFT studies, the difference in the CO_2_ adsorption properties and reaction pathway depending on the reduced and unreduced Ni based supported catalysts was discussed.

## Abstract 2

The effect of metal–support interaction and role of support on catalytic performances during Ni based CO_2_ methanation reaction were investigated.

## Graphical abstract

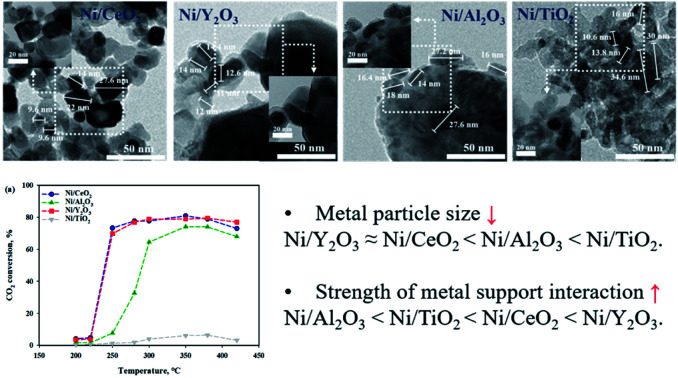

## 1 Introduction

The CO_2_ methanation reaction is important in industry because it produces methane and removes CO_2_. Among the CO_2_ conversion reactions using catalysts, the CO_2_ methanation reaction or so-called Sabatier reaction, is the most advantageous reaction.^1^ CO_2_ methanation is a simple reaction which makes it possible to integrate the transformation of methane into biogas through the process of anaerobic digestion to generate power, or into other industrial plants with CO_2_-rich exhaust gases.^2^ CO_2_ methanation is a highly exothermic reaction, and it can produce the heat. CO_2_ methanation also has a large equilibrium constant at a lower temperature, so that methane can be generated with a high conversion at lower temperatures; however, the conversion was still low due to kinetic limitation and catalyst performance. Catalytic performance is dependent on various parameters, such as the kinds of support, preparation methods, and the addition of promoters.^3^ Especially, many researchers have studied the effect of support to enhance the performance of the CO_2_ methanation reaction. The support has a significant effect on the redox properties, metal–support interaction, metal dispersion and adsorption properties.^4–6^ Many efforts have been made to develop Ni-based supported catalysts for low-temperature CO_2_ methanation.^7–11^ The CO_2_ methanation activity has been investigated using Ni-based catalysts deposited on various supports such as SiO_2_,^12^ α-Al_2_O_3_,^13^ MgO,^14^ ZrO_2_,^15^ Y_2_O_3_,^16^ CeO_2_,^17^ TiO_2_,^18^ β-zeolite,^19^ and their composite supports such as Ni/CeO_2_–ZrO_2_,^20^ Ni/γ-Al_2_O_3_–ZrO_2_–TiO_2_–CeO_2_,^21^ Ni/CeO_2_–Al_2_O_3_.^22^ Frontera *et al.*^23^ reported that the catalytic activity strongly effects on the characteristics of the support for Ni based catalysts. Le *et al.*^24^ founded that the high Ni dispersion and strong CO_2_ adsorption plays an important role in the high CO and CO_2_ methanation activities for Ni/CeO_2_ catalyst. Tada *et al.*^13^ reported that the Ni/CeO_2_ catalyst showed a high conversion compare to Ni/α-Al_2_O_3_. A large CO_2_ adsorption amount and high CO_2_ reduction ability could result in high CO_2_ conversion at a low temperature using a Ni/CeO_2_ catalyst. Muroyama *et al.*^16^ found that the Ni/Y_2_O_3_ catalyst exhibited a high CO_2_ conversion and CH_4_ yield compare to Ni/CeO_2_, Ni/Al_2_O_3_, Ni/ZrO_2_, Ni/La_2_O_3_ and Ni/Sm_2_O_3_ catalysts. They expected that the promotion of the decomposition of formate species would lead to high catalytic activity. Abello *et al.*^25^ have reported that the Ni–Al-activated catalyst prepared by a co-precipitation method exhibited a high CO_2_ conversion and stability at a high space velocity and highly loaded and dispersed small Ni nanoparticles (*ca.* 6 nm) dispersed over NiO–alumina by partial reduction of the mixed oxide. Vogt *et al.*^26^ reported that CO_2_ hydrogenation over Ni is considered to follow two steps; direct dissociation and H-mediated and they proved how structure sensitivity effects the mechanism of CO_2_ hydrogenation over Ni/SiO_2_. Zeolite and TiO_2_ supports are also used as Ni catalysts during CO_2_ methanation. Liu *et al.*^18^ reported that the 15 wt% Ni/TiO_2_ catalyst prepared by deposition–precipitation method showed excellent CO_2_ methanation activity (conversion: 96%; selectivity: 99%) at 260 °C. The good dispersion of Ni particles with high exposure of active sites, which may lead to enhanced exposure of active sites that facilitate the generation of surface-dissociated hydrogens. Recent reports have shown substantial improvement in the CO_2_ methanation rate by changing the support's properties and/or by the addition of promoters such as Ni–W–Mg,^27^ Ni–La,^28^ Ni–Cu or Fe/Al_2_O_3_ (ref. 29) catalysts. Many studies have examined the performance of highly active Ni-based catalysts supported on various metal oxides for CO_2_ methanation at low temperature. However, the effect of metal–support interaction and role of support on catalytic performances during Ni based CO_2_ methanation reaction is yet to be unraveled. In this work, CeO_2_, Y_2_O_3_, TiO_2_ and Al_2_O_3_ supports were selected as representative Ni catalysts and prepared by wet impregnation method. We investigated the catalytic activities of all catalysts for CO_2_ methanation and examined their physicochemical properties. Moreover, the adsorbed species on the reduced and unreduced catalyst surface were compared.

## 2 Experimental

### 2.1 Preparation of catalysts

Ni-Based catalysts supported on CeO_2_ (Sigma Aldrich Co.), α-Al_2_O_3_ (Alfa Aesar Co.), Y_2_O_3_ (Sigma Aldrich Co., St. Louis, MO, USA) and TiO_2_ (G-5, Cristal Global Co.) were prepared by a wet impregnation method. Nickel nitrate hexahydrate (Ni(NO_3_)_2_·6H_2_O; Sigma Aldrich Co.) was dissolved in distilled water at 80 °C. After impregnation, the moisture was removed at 80 °C using a rotary vacuum evaporator and then dried at 103 °C oven for 12 h. The samples were calcined at 400 °C for 2 h and then samples were reduced at 420 °C for 2 h with a 30% H_2_/N_2_. The obtained samples were grounded and sieved using a 40–50 mesh. Ni loading was fixed at 10 wt%.

### 2.2 Catalytic activity

**Fig. 1:**
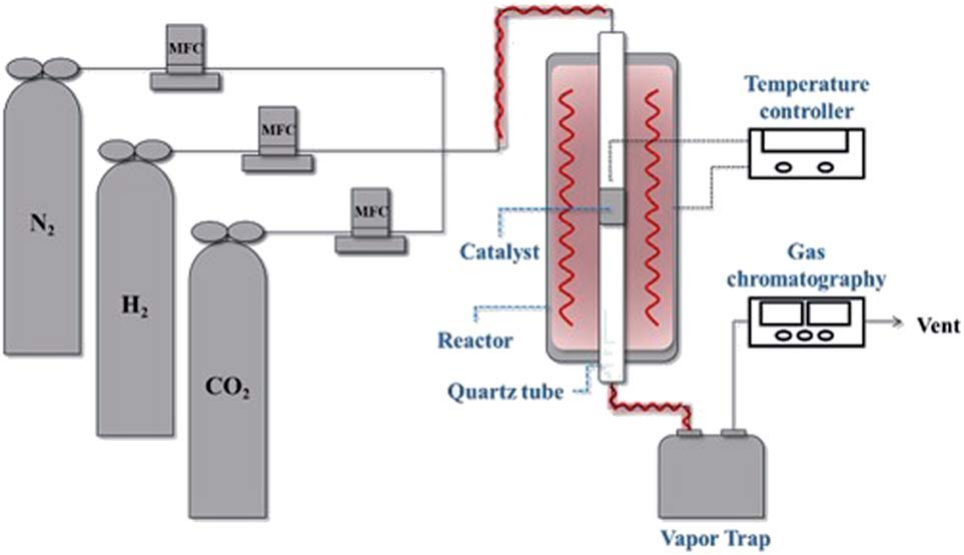
Schematic of the experimental fixed bed catalytic reactor for CO_2_ methanation.

The CO_2_ methanation experimental apparatus consisted of a continuous flow-type fixed-bed reactor comprising a quartz tube (inner diameter: 8 mm; height: 600 mm) and a catalytic bed (Fig. 1). To measure the gas temperature, another K-type thermocouple was installed at the top of the catalytic bed. Prior to the experiment, the catalysts were pretreated at 420 °C for 1 h with a 30% H_2_/N_2_ at a flow rate of 100 cm^3^ min^−1^. The feed gases comprised 16.67% CO_2_, 66.66% H_2_ and 16.67% N_2_. The total flow through the reactor was 120 cm^3^ min^−1^ and a space velocity of 14 400 l kg^−1^ h^−1^ was obtained. The outlet gas-supply pipe was made of stainless steel and wrapped with a heating band set at 180 °C to prevent water condensation. The concentrations of the reactants and products were measured as follows: the inlet and outlet gas concentration were analyzed using a gas chromatograph with a thermal conductivity detector (GOW-MAC, series 580). The CO_2_ conversion and yield can be calculated as the following:
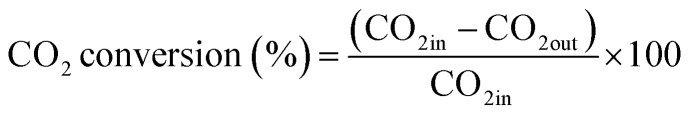

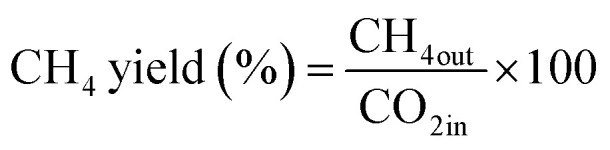

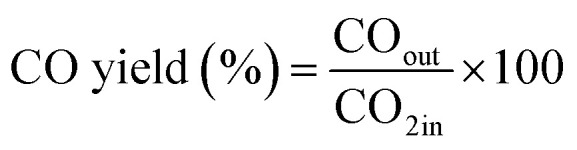


### 2.3 Characterization

The surface areas of Ni based catalysts were obtained by the Brunauer–Emmett–Teller (BET) equation using an ASAP 2010 instrument (Micrometrics). The Ni dispersion was evaluated by H_2_ chemisorption at 35 °C using a Micrometrics ASAP 2020 instrument (Micrometrics). All catalysts were reduced under a H_2_ airflow at 300 °C for 1 h and then cooled into 35 °C. X-Ray diffraction (XRD) analysis was measured on an X'Pert PRO MRD instrument (PANalytical) with a Cu Kα (*λ* = 1.5056 Å) radiation. Field emission-transmission electron microscope (TEM) analysis was carried out on a JEM-2100 F (JEOL) microscope (200 keV voltage). All samples were prepared by suspending an ultrasonicated catalyst powder in ethanol and placing the suspension on a Cu grid. For H_2_ chemisorption analysis, the catalysts were activated with 10% H_2_ at 300 °C for 0.5 h and then cooled to 50 °C and saturated with H_2_ pulses. The temperature-programmed reduction (TPR) of H_2_ was measured by 10% H_2_/Ar using 0.3 g of the catalyst at a total flow rate of 50 cm^3^ min^−1^. Before the H_2_ TPR measurement, the catalyst was pretreated in a flow of air at 400 °C for 0.5 h, followed by cooling to 50 °C. The catalyst was placed in dilute hydrogen, and the consumption of hydrogen was monitored using Autochem 2920 (Micrometrics) by increasing the temperature to 900 °C at a rate of 10 °C min^−1^. X-ray photoelectron spectroscopy (XPS) analysis was performed using an ESCALAB 210 (VG Scientific), and Al Kα monochromate (1486.6 eV) was used as an excitation source. Fourier-transform infrared (FT-IR) spectroscopy experiments were conducted in a diffuse reflection cell equipped with a CaF_2_ window using an FT-IR spectrometer (Nicolet IS 10, ThermoFisher), and diffuse reflectance (DR) 400 accessory was used. The spectra included 30 accumulated scans at resolutions of 4 cm^−1^, which were obtained using a mercury–cadmium–telluride (MCT) detector. To investigate the characteristics of CO_2_ adsorption and CO_2_ methanation reaction, the gas flowing over the samples pretreated by H_2_ and air were switched to CO_2_ or CO_2_ + H_2_ for 20 min at 200 and 300 °C.

## 3 Results and discussion

### 3.1 Catalytic activities

**Fig. 2:**
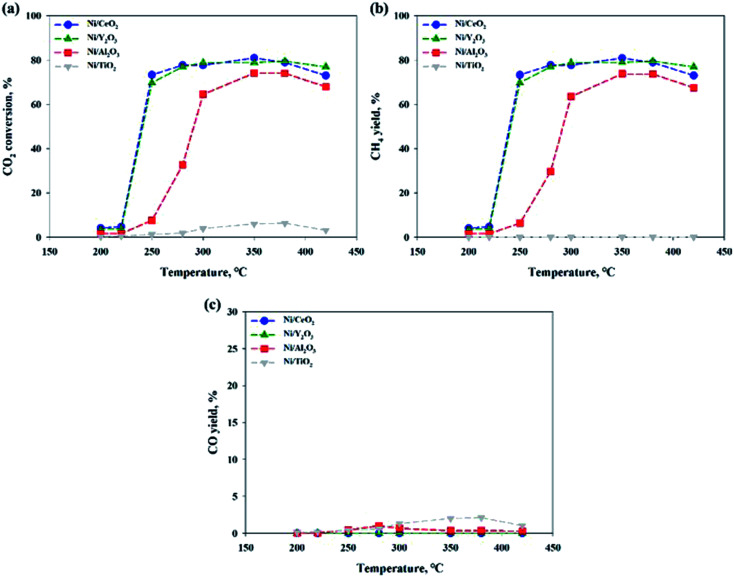
(a) CO_2_ conversions, (b) CH_4_ and (c) CO yield in CO_2_ methanation over 10 wt% Ni/support catalysts. CO_2_ : H_2_ : N_2_ = 1 : 4 : 1, GHSV: 14 400 l kg^−1^ h^−1^.

Fig. 2 shows the CO_2_ conversions of Ni-based catalysts supported on CeO_2_, TiO_2_, Y_2_O_3_ and Al_2_O_3_ at different reaction temperatures for the CO_2_ methanation reaction. Among the catalysts, the reduced Ni/CeO_2_ and Ni/Y_2_O_3_ catalysts exhibited the similar CO_2_ conversion over a range from 250 to 400 °C. For the reduced Ni/Al_2_O_3_ catalyst, the CO_2_ conversion decreased at 250–280 °C and then reached the maximal value at 350–380 °C. Especially, the Ni/TiO_2_ catalyst exhibited a very poor CO_2_ conversion at all temperature range. The CO_2_ to CH_4_ and CO conversions are also shown in Fig. 2(b) and (c). CO_2_ was fully converted to the CO (0.1–2.1%) in all temperature range for Ni/TiO_2_ catalyst. From the properties of the equilibrium conversion, the reverse water gas shift reaction is not favored at a low temperature. Thus, it is indicated that the CO_2_ should be able to convert CO on the reduction sites on a TiO_2_ support. Except for the Ni/TiO_2_ catalyst, CO yields were nearly zero at a reaction temperature under 300 °C, indicating that all CO_2_ conversion is mostly concordant with CO_2_ conversion to CH_4_. The order of catalytic activity regardless of the reaction temperature was as follows: Ni/CeO_2_ ≈ Ni/Y_2_O_3_ > Ni/Al_2_O_3_ > Ni/TiO_2_. It could be concluded that the reduced Ni/CeO_2_ and Ni/Y_2_O_3_ catalysts showed superior activity at a low temperature. Although catalytic activity of the Ni-based catalyst differs with operation conditions such as temperature, pressure, catalyst loading, and gas component, the general results showed similarities to previously reported studies by other researchers.^11,30,31^ Cai *et al.*^30^ reported that CO_2_ conversion of Ni/Ce_*x*_Zr_1−*x*_O_2_ catalyst showed the 72.21% at 390 °C. The higher reducibility of the Ce-rich supported highly-dispersed Ni catalyst was considered to be an important factor for long-term stability.^30,32,33^ However, the role of Ni dispersion in the stability of the Ni/CeO_2_ catalyst is difficult to elucidate in this work, and further studies will be necessary to fully address this point.

### 3.2 Physicochemical properties

**Fig. 3:**
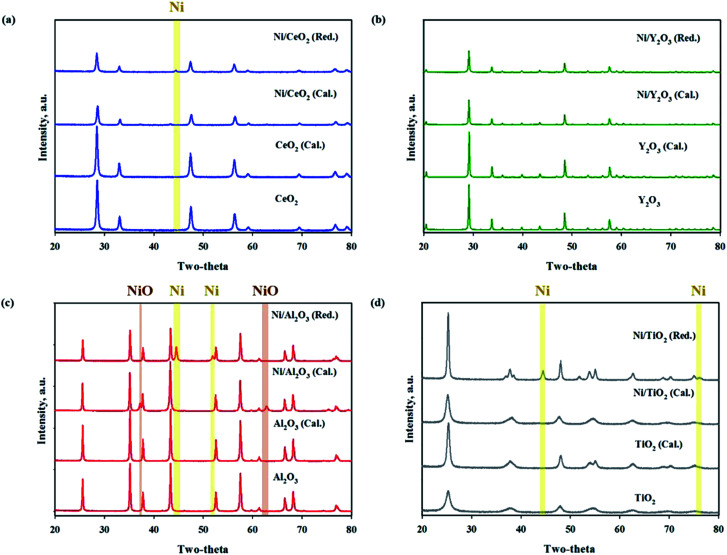
XRD patterns of metal oxide supports and reduced (red.) and unreduced (cal.) Ni supported catalysts ((a) Ni/CeO_2_, (b) Ni/Y_2_O_3_, (c) Ni/Al_2_O_3_, (d) Ni/TiO_2_).

**Fig. 4:**
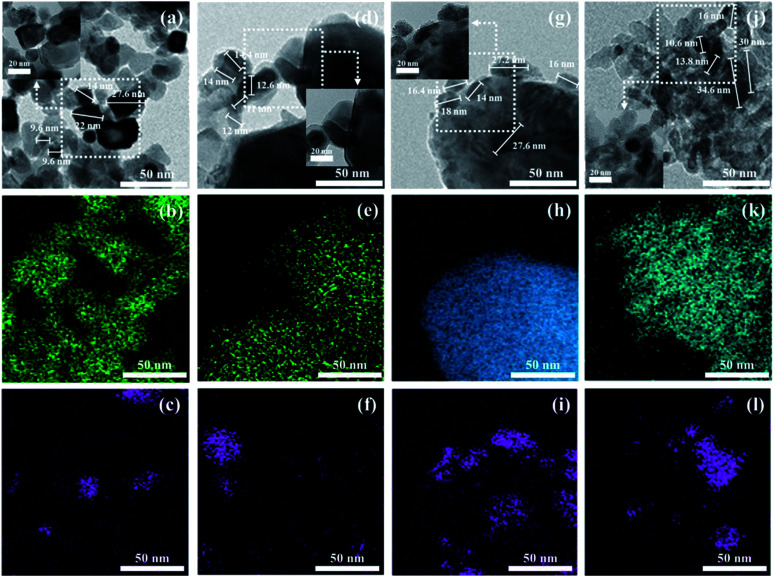
TEM and mapping images of Ni/support catalysts ((a) Ni/CeO_2_, (b) Ce, (c) Ni, (d) Ni/Y_2_O_3_, (e) Y_2_O_3_, (f) Ni, (g) Ni/Al_2_O_3_, (h) Al_2_O_3_, (i) Ni, (j) Ni/TiO_2_, (k) TiO_2_, (l) Ni).

The BET surface areas and metal dispersions of the reduced and unreduced samples are summarized in Table 1. The order of support's surface area was as follows: TiO_2_ > CeO_2_ > Al_2_O_3_ > Y_2_O_3_ (TiO_2_: 316.226, CeO_2_: 29.557, Al_2_O_3_: 7.375, Y_2_O_3_: 2.615 m^2^ g^−1^; not shown in the figure). It can be observed that the Ni/TiO_2_ catalyst had a much larger specific surface area than other catalysts. Zhang *et al.*^34^ showed that the catalyst's specific surface area is not directly related to the catalytic activity in CO_2_ methanation for Ni-based catalysts. Our results were in good agreement with previously mentioned evidence from the literature. The reduced Ni/TiO_2_ catalyst had the lowest value of Ni dispersion (0.0116%). The reduced Ni/Y_2_O_3_ catalyst showed the highest metal dispersion (2.7883%) of all the unreduced Ni-based catalysts. Reduction degree of surface area and metal dispersion for Ni based catalysts with heat treatment by air or H_2_ can be attributed to complex interdependency on metal–support interactions. This would suggest that a decrease of metal dispersion by reduction treatment could be explained by a strong interaction between Ni and oxide support. The order of strength of the metal and support interaction was as follows: Ni/Al_2_O_3_ < Ni/TiO_2_ < Ni/CeO_2_ < Ni/Y_2_O_3_. Fig. 3(a)–(d) shows the XRD patterns of raw supports, calcined supports, calcined Ni based catalysts and calcined Ni based catalysts. The XRD pattern of Ni consists of three main peaks at 45°, 53°, and 76° corresponding to (111), (200), and (220) planes, respectively. The XRD pattern of NiO consists of two main peaks at 37° and 62° corresponding to (111) and (220) planes, respectively.^35^ For the TiO_2_ powder, the main peaks were observed to be at 2*θ* = 25.3, 37.1 and 47.5°, corresponding to typical anatase TiO_2_ peaks. The sharp peaks were observed for the crystallite TiO_2_ structure when the amorphous TiO_2_ powder was calcined at a temperature of 400 °C. NiO peaks were not observed in calcined Ni/TiO_2_ catalyst. It is interesting to note that Ni crystallite main peaks were detected at 2*θ* = at 44.5, 51.8 when using the reduced Ni/TiO_2_ catalyst. The release of bonding oxygen atoms within Ni–O–Ti by H_2_ reduction will help to move the Ni particles, then the Ni particles are easily agglomerated. Ni and NiO peaks were observed in both reduced and unreduced Ni/Al_2_O_3_ catalysts. Unlike the Ni/TiO_2_ catalyst, it confirmed that Ni and NiO particles were agglomorated by heat treatment regardless of the migration of oxygen atom for Ni supported on irreducible Al_2_O_3_. CeO_2_ and Y_2_O_3_ support had quite low surface area. Nevertheless, the Ni and NiO peaks were not observed in reduced and unreduced Ni/Y_2_O_3_ catalyst and very low Ni and NiO peaks were observed in reduced and unreduced Ni/CeO_2_ catalyst, which is related to strong metal–support interactions (SMSI effect). This phenomenon is in agreement with results from previous metal dispersion. TEM analysis was conducted to estimate the Ni particle sizes of Ni based catalysts. The TEM and mapping images of the reduced Ni catalysts are presented in Fig. 4(a)–(l). It could be seen that the Ni/Y_2_O_3_ mean particle size of Ni was 12.8 nm, while that of Ni/CeO_2_, Ni/Al_2_O_3_ and Ni/TiO_2_ were 16.5 nm, 19.9 nm and 21 nm, respectively. Vogt *et al.*^25^ investigated the particle size effect of Ni/SiO_2_ catalysts prepared by homogeneous deposition precipitation and co-precipitation with different Ni loadings (1–60 wt%). It was concluded that the Ni based CO_2_ methanation is structure sensitive from 1–7 nm for Ni/SiO_2_ catalyst. Many studies have attempted to enhance the Ni dispersion by increasing H_2_ adsorption as active sites.^11,36–39^ In this study, the 10 wt% Ni-based catalysts prepared by the impregnation method have relatively large Ni particle size of 12.8–21 nm, but it has low temperature CO_2_ methanation activity. In the case of Ni/Al_2_O_3_ and Ni/TiO_2_ catalyst, it was confirmed that the difference in activity at high temperature (280–420 °C) was clearly displayed despite the similar particle size and metal dispersion. The fact that the Ni particles play an important role in the adsorption of hydrogen as active sites, but other factors such as CO_2_ adsorption characteristics and oxygen transfer by hydrogen can influence the catalytic performance.

**Table 1:** BET surface area and metal dispersion of reduced and unreduced Ni/metal oxide catalysts

	Surface area (m^2^ g^−1^)		Metal dispersion^a^ (%)	
	Unreduced	Reduced	Unreduced	Reduced
Ni/CeO_2_	29.907	22.845	4.932	1.823
Ni/Y_2_O_3_	16.794	21.233	3.438	2.788
Ni/Al_2_O_3_	9.867	8.698	2.323	0.027
Ni/TiO_2_	121.74	43.606	0.067	0.012

a Calculated by H_2_ pulse chemisorption.

**Fig. 5:**
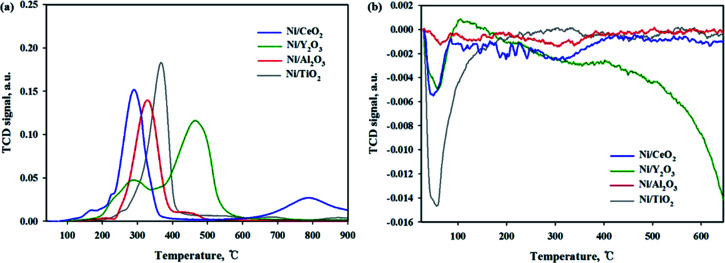
(a) H_2_-TPR and (b) CO_2_ TPO profiles of Ni/metal oxide catalysts.

The reducibility of Ni-based catalysts was investigated by a H_2_-temperature programmed reduction, and the profiles are shown in Fig. 5(a). A similar H_2_-TPR result for the Ni/CeO_2_ catalyst was previously reported.^13,17,40^ Zhou *et al.*^17^ reported that three reduction peaks can be seen at around 220 °C, 280–330 °C and 380 °C. The first low temperature peak attributed to the reduction of highly dispersed NiO species. The second peak attributed to the reduction of NiO species on the subsurface of the Ni/CeO_2_ catalyst and highly dispersive NiO species. The last peak can be assigned to the reduction of bulk NiO species. Tada *et al.*^13^ reported that the two reduction peaks can be observed at 340 and 420 °C, which were attributed to the reduction of NiO on CeO_2_ at about 400 °C. Ding *et al.*^40^ also reported that the main reduction peaks appeared at 350–450 °C; these peaks were attributed to the reduction of NiO. In this study, the Ni/CeO_2_ catalyst exhibited four-hydrogen-consumption maximum peaks at 165 °C, 225 °C, 290 °C and 785 °C, which were attributed to highly dispersive NiO species or Ni(OH)_2_, NiO species on the subsurfaces, Ni–O–Ce species, and bulk CeO_2_ reduction peaks, respectively. It should be noted that all the reduction peaks are shifted to a lower temperature compared to previously reported studies. It can be suggested that, although the catalytic composition was the same, the reduction trend can differ, depending on the preparation conditions *e.g.*, the kinds of precursor, calcination temperature/time, and metal loadings. For the Ni/Al_2_O_3_ catalyst, two reduction peaks were observed at 250–400 °C and 400–500 °C. The first peak is assigned to the free Ni species and second peak is attributed to the Ni species combined with the Al_2_O_3_ support (Ni–O–Al).^40^ Evidence from the literature showed that the last reduction peak can be observed for the Ni/Al_2_O_3_ catalyst in the high-temperature region (750–850 °C), suggesting a stronger interaction between NiO and the Al_2_O_3_ support.^21^ But this peak was not observed in this sample; this might be due to a low calcination temperature. For the Ni/Y_2_O_3_ catalyst, the two broad reduction peaks were observed in the 200–350 °C and 370–600 °C ranges, respectively. The first peak indicate the existence of a interaction between NiO and the Y_2_O_3_ support and the second reduction peak is attributed to the bulk Y_2_O_3_ support. The Ni/TiO_2_ catalyst exhibited only one broad hydrogen consumption peak at 250–420 °C, which was attributed to the Ni species combined with the TiO_2_ support (Ni–O–Ti). It is expected that the 10 wt% Ni based catalysts may have a metallic Ni form at 420 °C of reduction temperature. According to the H_2_ TPR and activity test results of all catalysts, enhancement of the CO_2_ conversion could be mainly due to the higher amount of NiO species able to be reduced at low temperature. CO_2_ temperature-programmed oxidation (TPO) was performed to observe the CO_2_ conversion for reduced catalysts at 420 °C as shown in Fig. 5(b). For the reduced Ni/TiO_2_ catalyst, the one huge CO_2_ consumption peak was observed in the 50–150 °C. This result indicated that the reduced Ni/TiO_2_ catalyst was able to accept oxygen by introduction of CO_2_ on reducible sites. However, on the Ni surface H, adsorption is difficult due to low Ni dispersion, which makes it difficult to react the CO_2_ methanation. The reduced Ni/CeO_2_ and Ni/Y_2_O_3_ catalysts exhibited the similar CO_2_ consumption peak at 50–100 °C. For the reduced Ni/Al_2_O_3_ catalyst, the CO_2_ consumption peak was not observed at all temperature range, it may be concluded that the CO_2_ molecules cannot be dissociated on reduced Ni/Al_2_O_3_ catalyst. Among the catalysts used in this study, the Ni/CeO_2_ and Ni/Y_2_O_3_ catalyst had the higher dispersion and enhanced reducibility of Ni particles, as well as strong metal and support interactions, which could become an important factor for the catalytic activity during low-temperature CO_2_ methanation.

### 3.3 *In situ* DRIFTS

**Fig. 6:**
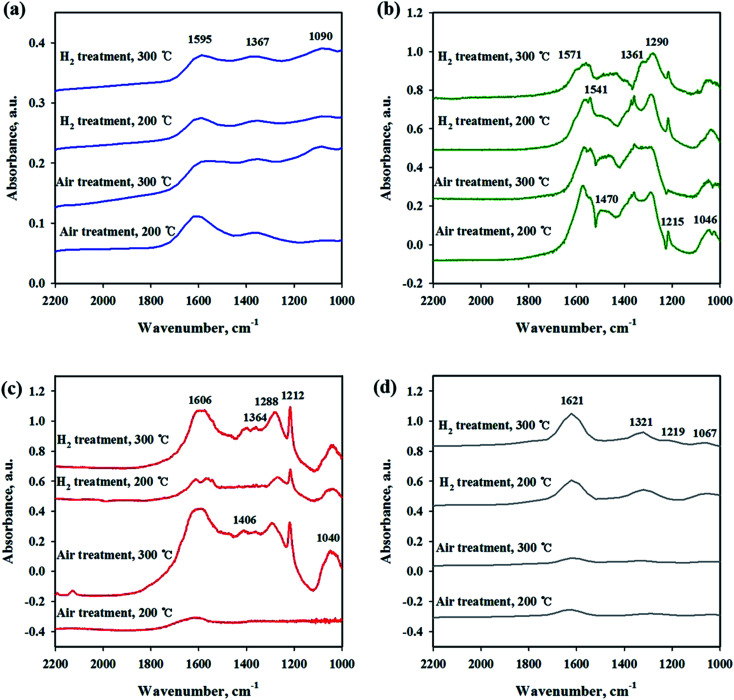
*In situ* DRIFTS studies of CO_2_ adsorption on Ni/metal oxide catalysts with different pretreatment and temperature conditions. (a) Ni/CeO_2_, (b) Ni/Y_2_O_3_, (c) Ni/Al_2_O_3_ and (d) Ni/TiO_2_.

**Fig. 7:**
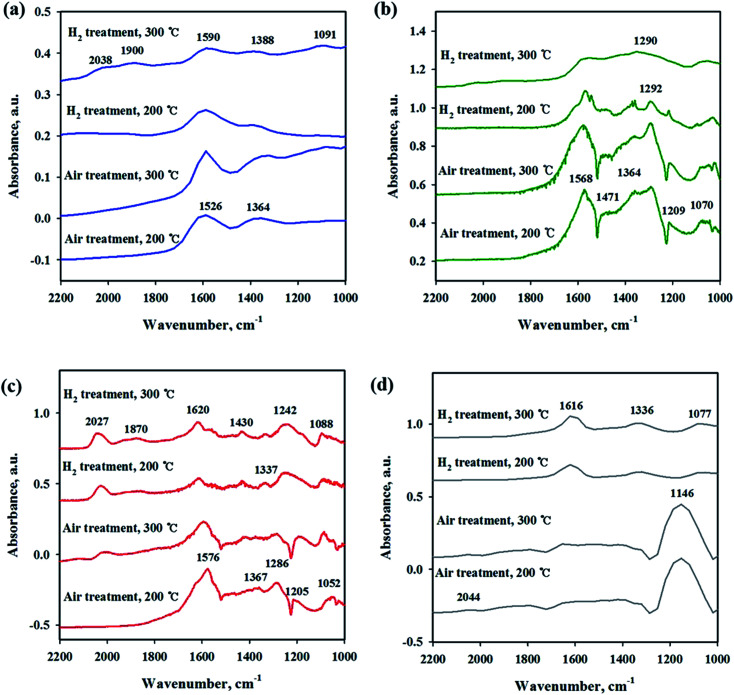
*In situ* DRIFTS studies of CO_2_ methanation (CO_2_ + H_2_) on Ni/metal oxide catalysts with different pretreatment and temperature conditions. (a) Ni/CeO_2_, (b) Ni/Y_2_O_3_, (c) Ni/Al_2_O_3_ and (d) Ni/TiO_2_.

To investigate the interaction of CO_2_ with catalysts, *in situ* DRIFT studies were performed on the reduced and unreduced catalysts at 200 and 300 °C as shown in Fig. 6. Fig. 6(a) shows the CO_2_ adsorption profile on the Ni/CeO_2_ catalyst surface. The assignment of CO_2_ adsorption bands for the Ni/Ce_0.5_Zr_0.5_O_2_ catalyst was performed by reported in the previous literature.^41,42^ The spectra of CO_2_ adsorption on Ni/CeO_2_ are similar to those of the Ni/Ce_0.5_Zr_0.5_O_2_ catalyst. The band observed at 1595 cm^−1^ can be assigned to formate species and the bands centered at 1367 cm^−1^ was assigned to monodentate carbonate. These two main bands were still maintained at 300 °C, implying that the formation of formate and monodentate carbonate species are favored at high temperature, as the main intermediate during direct hydrogenation of CO_2_.^41,43–46^ The reduced and unreduced Ni/CeO_2_ catalysts exhibited similar CO_2_ adsorption bands, regardless of calcination or reduction treatments, indicating that the CO_2_ adsorption properties on Ni/CeO_2_ catalyst may depend on the CeO_2_ support, not NiO and metallic Ni species. CO_2_ adsorption bands were compared for Ni/Y_2_O_3_ and Ni/Al_2_O_3_ in Fig. 6(b) and (c). The main adsorption bands were detected at 1541, 1290, 1215 and 1046 cm^−1^ (formate), and 1571 cm^−1^ (bidentate carbonate) for Ni/Y_2_O_3_ catalyst and at 1606, 1406 and 1364 cm^−1^ (formate), and 1364 cm^−1^ (bidentate carbonate) for Ni/Al_2_O_3_. The formate bands were maintained at 300 °C for Ni/Al_2_O_3_ and Ni/Y_2_O_3_ catalysts. The intensity of main peaks increased with higher temperature (300 °C) for Ni/Al_2_O_3_ catalyst. In Fig. 6(d), the CO_2_ adsorption bands were observed for the Ni/TiO_2_ catalyst. The bands centered at 1219 and 1067 cm^−1^ were attributed to monodentate species and the bands centered at 1620 and 1219 cm^−1^ were assigned to hydrogen carbonate peaks. The intensity of CO_2_ absorbed peaks decreased at calcined catalyst while these peaks increased with H_2_ reduction treatment, indicating that a small part of catalytic sites become active by surface OH groups, and CO_2_ adsorption peaks are increased. Fig. 7 shows the CO_2_ adsorption profiles of all reduced and unreduced catalysts in the CO_2_–4H_2_ atmosphere as a function of different temperatures. The two main bands attributed to formate and monodentate carbonate were still maintained at 200 and 300 °C for the calcined Ni/CeO_2_ catalyst when introducing CO_2_ and H_2_ (Fig. 7(a)). These peaks were maintained at the reaction temperature of 200 °C for reduced Ni/CeO_2_ catalyst but the intensity of peaks decreased obviously at reaction temperature of 300 °C and the two bands were detected at 1900 and 2038 cm^−1^ which was assigned bridged CO and linear CO bands adsorbed on Ni, respectively.^47^ This result indicated that CO_2_ was readily dissociated to CO and it does participate in the CO_2_ methanation reaction at high temperature for Ni/CeO_2_ catalyst. Aldana *et al.*^44^ reported that methanation proceeds through formate species originated from the hydrogenation of carbonates and CO was formed by a redox cycle on reduced ceria support. At 300 °C, only monodentate carbonate bands well remained. This result indicated that the monodentate carbonates are not easily hydrogenated; this result was in good agreement with Pan *et al.*^41^ As shown in Fig. 7(b), although hydrogen is injected, the main formate and bidentate carbonate bands were still maintained at 200 and 300 °C for the calcined Ni/Y_2_O_3_ catalyst. The small and broad formate bands observed at 300 °C for the reduced Ni/Y_2_O_3_ catalyst and the two small bands were detected at 1900 and 2038 cm^−1^ which was assigned bridged CO and linear CO bands adsorbed on Ni, respectively, it form a similar pattern with reduced Ni/CeO_2_ catalyst. This formation of active species as formate leads to higher catalytic activity at high temperature. It is expected that sufficient reduction pretreatment of reducible supports such as CeO_2_ and Y_2_O_3_ supported catalysts efforts on CO_2_ decomposition into CO which is an crucial step in CO_2_ conversion into CH_4_ at high temperature and thus catalytic activity using Ni/CeO_2_ and Ni/Y_2_O_3_ catalysts is enhanced.^13^ In Fig. 7(c), the CO_2_ absorption bands were still maintained at 200 °C for the calcined Ni/Al_2_O_3_ catalyst, it forms a similar pattern with CO_2_ adsorption pattern as shown in Fig. 6(c). CO_2_ methanation is not progressed in this low temperature for Ni/Al_2_O_3_ catalyst. The similar bands were detected at 300 °C for the calcined Ni/Al_2_O_3_ catalyst, but the small band observed at 2027 cm^−1^ which was assigned linear CO bands adsorbed on Ni. It is known from the H_2_ TPR results that the release of bonding oxygen atoms within Ni–O–Al by H_2_ reduction at 300 °C, CO_2_ was readily dissociated to CO on reduction active site for the calcined Ni/Al_2_O_3_ catalyst. The spectra for the reduced Ni/Al_2_O_3_ catalyst exhibited small overlapped bands of formate species and the intensity of bridged CO and linear CO peaks centered at 1870 and 2027 cm^−1^ increased. This result indicated that CO_2_ was readily dissociated to CO and it does participate in the CO_2_ methanation reaction at high temperature for reduced Ni/Al_2_O_3_ catalyst. The Ni/TiO_2_ catalyst showed a different adsorption bands than other catalyst as shown in Fig. 7(d). The intensity of broad hydrogen carbonate peak centered at 1000–1300 cm^−1^ increased for calcination Ni/TiO_2_ catalyst and the two small bands were observed at 1336 and 1077 cm^−1^ which was assigned monodentate peaks regardless of reaction temperature. The amount of CO_2_ adsorbed onto Ni/TiO_2_ was much larger than that onto other catalyst, but CO_2_ methanation reaction cannot occur regardless of reaction temperature. Due to the low Ni dispersion, it can be inferred that adsorbed monodentate and hydrogen carbonate species are difficult to react with adsorbed hydrogen atoms on active metallic Ni site to form methane so that CO_2_ methanation reaction does not take place at all temperatures. Numerous studies about reaction mechanism of CO_2_ methanation have been investigated. The reaction mechanisms are normally classified into two reaction pathways. One involves CO_2_ conversion to CO as intermediate, which then follows the same mechanism as CO methanation.^48^ The other one involves formate and carbonate as main intermediate, which directly hydrogenate without forming CO.^44^ From the results in this study, it was assumed that the methanation reaction of CO_2_ will follow the first mechanism for Ni/Al_2_O_3_ catalyst and formate and carbonate as main intermediate mechanism at low temperature (200 °C) for Ni/Y_2_O_3_ and Ni/CeO_2_ catalysts. However, the methanation reaction of CO_2_ will follow the first mechanism at high temperature (300 °C) for Ni/Y_2_O_3_ and Ni/CeO_2_ catalysts. These different mechanisms might be one of the reasons why the role of supports. The Ni supported irreducible Al_2_O_3_ catalyst favors the CO_2_ conversion to CO and then follows the same mechanism as CO methanation. While carbonate and formate species were found to be the main intermediate on surface oxygen vacancy site such as Ce^3+^ sites, which could enhance the catalytic activity at low temperature compare to Ni/Al_2_O_3_ catalyst.^3^ The carbonate and formate species were mainly present as an adsorption intermediate on surface oxygen vacancy site (Ce^3+^ sites), while the CO species were found to be the main adsorption intermediate for the irreducible Al_2_O_3_ supported Ni catalyst. According to TPR and DRIFT results, the interaction between Ni and Ce or Y may facilitate the formation of the OH groups on the Ni–OH or Y–OH, Ce–OH bonds. The dissociated hydrogen atoms on the Ni metal spillover onto the ceria or yttria support surface, and undergo surface diffusion at low temperature by introduction of hydrogen. Superior reducibility of both Ni-rich surface species and CeO_2_ support by strong interaction between Ni and CeO_2_ or Y_2_O_3_ supports are important factor and then weakly adsorbed CO_2_ species such as formate and carbonate on the surface oxygen vacancy site easily reacted with hydrogen dissociation on the metal followed by spillover at low temperature.

## 4 Conclusions

The Ni/CeO_2_ and Ni/Y_2_O_3_ catalysts show highly enhanced catalytic activity for low-temperature CO_2_ methanation, as compared with the Ni/Al_2_O_3_ and Ni/TiO_2_ catalysts. The increase in the low-temperature CO_2_ methanation activity can be directly correlated with an enhancement in the reducibility and small Ni particle size of the Ni/CeO_2_ and Ni/Y_2_O_3_ catalyst, which is caused by a strong interaction between reduced CeO_2_ or Y_2_O_3_ oxide support and Ni. When Ni species are dispersed on “reducible” oxides, such as CeO_2_ and Y_2_O_3_, carbonate and formate species were mainly present as an adsorption intermediate on the surface oxygen vacancy site such as Y^(3−*x*)+^ or Ce^3+^ sites at low temperature, which plays a key role in enhancing the low catalytic activity. Due to the low Ni dispersion of Ni/TiO_2_ catalyst, it can be concluded that adsorbed CO_2_ species are difficult to react with hydrogen atoms on active metallic Ni site to form methane. When the Ni species were dispersed on “irreducible” oxides, species such as Al_2_O_3_ and CO were found to be the main adsorption intermediates. Overall, CeO_2_ and Y_2_O_3_ supports are promising support for the Ni-based catalyst and further improvement in low-temperature CO_2_ methanation activity can be made by modification of the mixed CeO_2_ and Y_2_O_3_ support with small Ni particle size and oxygen vacancies.

## Author contributions

Ye Hwan Lee: conceptualization, writing – original draft. Jeong Yoon Ahn: experiment and evaluation. Dinh Duc Nguyen: data curation, validation, formal analysis. Soon Woong Chang: writing – review & editing. Sung Su Kim: writing – review & editing. Sang Moon Lee: supervision, project administration.

## Conflicts of interest

There are no conflicts to declare.

## Supplementary Material
